# Anti-Alzheimer’s Molecules Derived from Marine Life: Understanding Molecular Mechanisms and Therapeutic Potential

**DOI:** 10.3390/md19050251

**Published:** 2021-04-28

**Authors:** Md. Tanvir Kabir, Md. Sahab Uddin, Philippe Jeandet, Talha Bin Emran, Saikat Mitra, Ghadeer M. Albadrani, Amany A. Sayed, Mohamed M. Abdel-Daim, Jesus Simal-Gandara

**Affiliations:** 1 Department of Pharmacy, Brac University, Dhaka 1212, Bangladesh; tanvir_kbr@yahoo.com; 2 Department of Pharmacy, Southeast University, Dhaka 1213, Bangladesh; 3 Pharmakon Neuroscience Research Network, Dhaka 1207, Bangladesh; 4 Research Unit, Induced Resistance and Plant Bioprotection, USC INRAe 1488, SFR Condorcet FR CNRS 3417, Faculty of Sciences, University of Reims Champagne-Ardenne, P.O. Box 1039, CEDEX 2, 51687 Reims, France; philippe.jeandet@univ-reims.fr; 5 Department of Pharmacy, BGC Trust University Bangladesh, Chittagong 4381, Bangladesh; talhabmb@bgctub.ac.bd; 6 Department of Pharmacy, Faculty of Pharmacy, University of Dhaka, Dhaka 1000, Bangladesh; saikatmitradu@gmail.com; 7 Department of Biology, College of Science, Princess Nourah bint Abdulrahman University, Riyadh 11474, Saudi Arabia; gmalbadrani@pnu.edu.sa; 8 Zoology Department, Faculty of Science, Cairo University, Giza 12613, Egypt; amanyasayed@sci.cu.edu.eg; 9 Pharmacology Department, Faculty of Veterinary Medicine, Suez Canal University, Ismailia 41522, Egypt; abdeldaim.m@vet.suez.edu.eg; 10 Nutrition and Bromatology Group, Department of Analytical and Food Chemistry, Faculty of Food Science and Technology, University of Vigo, Ourense Campus, E32004 Ourense, Spain

**Keywords:** Alzheimer’s disease, Aβ aggregation, tau phosphorylation, bryostatin-1, marine life, marine drugs

## Abstract

Alzheimer’s disease (AD) is a devastating neurodegenerative disease and the most common cause of dementia. It has been confirmed that the pathological processes that intervene in AD development are linked with oxidative damage to neurons, neuroinflammation, tau phosphorylation, amyloid beta (Aβ) aggregation, glutamate excitotoxicity, and cholinergic deficit. Still, there is no available therapy that can cure AD. Available therapies only manage some of the AD symptoms at the early stages of AD. Various studies have revealed that bioactive compounds derived from marine organisms and plants can exert neuroprotective activities with fewer adverse events, as compared with synthetic drugs. Furthermore, marine organisms have been identified as a source of novel compounds with therapeutic potential. Thus, there is a growing interest regarding bioactive compounds derived from marine sources that have anti-AD potentials. Various marine drugs including bryostatin-1, homotaurine, anabaseine and its derivative, rifampicins, anhydroexfoliamycin, undecylprodigioisin, gracilins, 13-desmethyl spirolide-C, and dictyostatin displayed excellent bioavailability and efficacy against AD. Most of these marine drugs were found to be well-tolerated in AD patients, along with no significant drug-associated adverse events. In this review, we focus on the drugs derived from marine life that can be useful in AD treatment and also summarize the therapeutic agents that are currently used to treat AD.

## 1. Introduction

Alzheimer’s disease (AD) is widely known as the most common cause of dementia, and AD is most frequently observed in older individuals [1,2]. Characteristics of AD include behavioral disturbances, neuronal death, memory loss, cognitive deficit, and cholinergic dysfunction. AD pathogenesis includes complex processes and a deficit of the neural pathways associated with memory function [3]. Early-onset AD has been detected in individuals over 65 years old. Nonetheless, over 90% of diagnosed cases are linked with the late-onset of AD, which is commonly observed in individuals over 65 years old [4]. On the other hand, preselinin 1 (*PSEN1*) mutation (P117L) is linked to familial AD (FAD) and can cause death of as young as 28 years old [5]. It has been reported that early-onset AD development is linked with various genetic mutations, particularly in amyloid precursor protein (*APP*), *PSEN1*, and preselinin 2 (*PSEN2*) genes [6]. Dysregulated expression of these genes might be present in around 5–10% of diagnosed cases of early-onset AD [4,6]. Indeed, apolipoprotein E (APOE) polymorphic alleles play a significant role in the development of early-onset and late-onset AD [7,8]. In addition, the presence of *APOE4* alleles is linked with an elevated risk of cerebral amyloid angiopathy and age-associated cognitive deficit during normal aging [9]. 

Major neuropathological features of AD include nerve cell death, intracellular neurofibrillary tangles (NFTs), and extracellular amyloid plaques [10,11,12,13,14]. Sequential APP cleavage takes place via two pathways, including the amyloidogenic pathway and the non-amyloidogenic pathway [15]. The amyloid plaques are made of amyloid beta (Aβ), which is generated by the amyloidogenic APP cleavage. In this pathway, APP is cleaved via β-secretase (BACE1) and subsequently via γ-secretase to generate Aβ [14]. It has been revealed that there is a link between FAD mutations and increased ratio of Aβ42/40 [16,17], which indicates that increased Aβ42 levels (as compared to Aβ40) play a crucial role in AD pathogenesis, possibly via providing the core for Aβ assembly into amyloidogenic plaques, fibrils, and oligomers [18,19]. In the elderly, Aβ accumulation might take place due to the change in APP cleavage. It was reported that an excessive level of age-linked acetylation of the α-secretase gene might reduce non-amyloidogenic APP processing [20]. In early AD brain tissue, increased BACE1 action was found to elevate amyloidogenic APP processing [21,22]. Monomers of Aβ progressively aggregate into fibrils, oligomers, and insoluble amyloid plaques [10]. NFTs are composed of hyperphosphorylated tau, and these NFTs are known as the histopathological hallmark of AD [23]. Tau can mediate the stabilization of microtubules under normal conditions. In contrast, when tau is hyperphosphorylated, it can accumulate into tangles made of paired helical filaments [11]. It is suggested by the amyloid cascade hypothesis that Aβ accumulation dysregulates neuronal and synaptic function, which can mediate the intracellular environment for the formation of NFTs, eventually resulting in loss of neurons and further deterioration of neurotransmitter activity [10]. 

Pharmacologically active substances are extracted from marine organisms, and these substances are developed into suitable forms for use in humans. Indeed, the ocean serves as a source of numerous bioactive substances; however, the ocean is still largely unexploited [24,25,26,27]. It was revealed through the isolation of soft corals that marine organisms can be an important source for novel drugs containing novel chemical structures and an increased level of therapeutic value. Moreover, the marine ecosystem is a significant source for discovering effective therapeutic agents, and marine organisms are associated with half of the Earth’s biodiversity [28,29]. The occurrence of new infections, metabolic disorders, and the increased rate of lifestyle and aging-associated diseases suggest that there is value in the constant exploration for more effective and highly selective drugs, utilizing both modern and traditional methods for designing and developing novel drugs. Various important drugs are abundantly present in microorganisms, invertebrates, and algae [30]. Contemporary technologies have opened massive research areas for the isolation of bioactive substances from seas and oceans [31]. Various sessile marine invertebrates including tunicates, bryozoans, and sponges have already served as a major source of several marine-derived secondary metabolites that have significant therapeutic potential. The occurrence of multiple novel metabolites, including bryostatin, suggests that the ocean is a rich source of numerous important drug leads [32,33]. In a phase IIa clinical trial, concentrations of bryostatin reached the maximum level at one to two hours from the onset of infusion [34]. Along with the maximum bryostatin levels in the blood, a rise in the concentration of PBMC protein kinase C epsilon (PKCɛ) was measured within one hour after the start of infusion. It was also reported that bryostatin elevated Mini-Mental State Examination (MMSE) scores. Moreover, bryostatin was found to be well-tolerated, and no drug-associated adverse reactions were reported in AD individuals. In addition, animal studies revealed that bryostatin elevated concentrations of postsynaptic density protein 95 (PSD-95) and brain-derived neurotrophic factor (BDNF) and effective PKCɛ activation. Collectively, these findings indicated bryostatin 1 as an effective AD treatment [34]. 

In this review, we focus on the drugs derived from marine organisms that can be useful in AD treatment. Furthermore, we also summarize the therapeutic agents that are currently used to treat AD. 

## 2. Current Alzheimer’s Drug Therapy

Although the number of AD individuals is growing, there are only five approved drugs currently in use for AD treatment in the United States [35]. In the European Union, four out of the five standard-of-care treatments for AD include an antagonist of N-methyl-D-aspartate receptor (memantine) and cholinesterase inhibitors (rivastigmine, galantamine, and donepezil) [36,37,38,39]. Unfortunately, no currently available drugs can stop or alter AD progression; rather these drugs improve AD symptoms for a limited time period and in a limited number of patients [40,41]. Tacrine was the first FDA-approved drug (in 1993), and it is currently discontinued owing to its liver toxicity. Donepezil was then approved by the FDA in 1996. Memantine and galantamine were approved by the FDA in 2003 and 2004, respectively [42]. Rivastigmine finally received FDA approval in 2006 [43]. The fifth therapeutic option containing a fixed-dose combination of memantine and donepezil received approval in 2014 to treat patients with moderate-severe AD who were receiving a stable therapy with donepezil [44,45,46]. 

Over the past 15 years, most of the drug candidates under development have failed. AD is a devastating neurodegenerative disease, but there is still no effective drug that can cure this disease. However, there is a growing understanding regarding AD complexity, its diverse pathogenetic modes, and the dynamic interaction between the constituents that contribute to AD [47]. Furthermore, promising findings obtained from vaccination trials in transgenic animal models have encouraged the development of immunotherapeutic agents for AD treatment [48]. Various monoclonal and polyclonal antibodies have been developed against Aβ and are currently in clinical trials. Novel experimental methods, including single-chain variable fragment antibodies, antibodies recognizing specific conformational epitopes, or intrabodies provide optimism for further drug development for AD [49]. Therefore, more studies considering the complex nature of AD are required in order to develop effective and novel anti-AD therapeutic molecules. 

## 3. Therapeutic Potential of Marine-Derived Anti-Alzheimer’s Molecules

### 3.1. Bryostatin-1 

#### 3.1.1. Preclinical Evidence 

**Figure 1:**
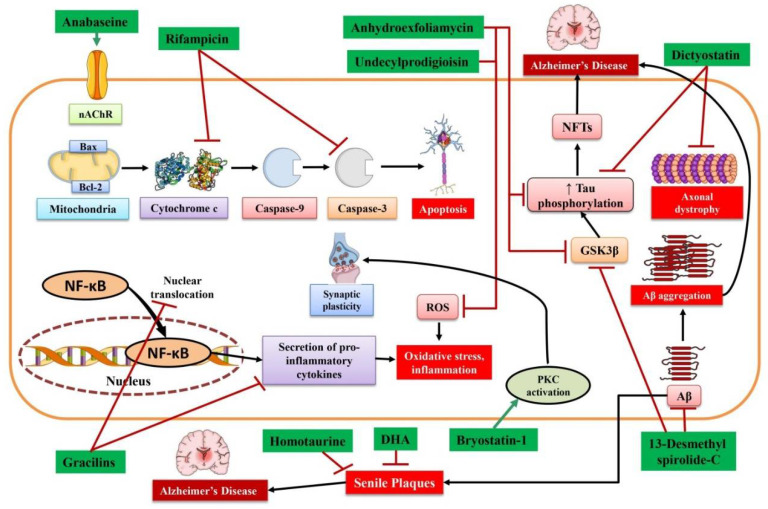
Mechanism of action of various marine-derived bioactive compounds in Alzheimer’s disease. Aβ, amyloid beta; Bax, Bcl-2-associated X protein; Bcl-2, B-cell lymphoma 2; DHA, docosahexaenoic acid; GSK3β, glycogen synthase kinase 3 beta; nAChR, nicotinic acetylcholine receptor; NFTs, neurofibrillary tangles; NF-κB, nuclear factor-kappa B; PKC, protein kinase C; ROS, reactive oxygen species.

Bryostatin 1 is a macrocyclic lactone isolated from the marine invertebrate *Bugula neritina* [50]. It is a potent activator of protein kinase C family members, along with nanomolar potency for PKC1ε and α isotypes. Furthermore, bryostatin 1 mediates PKC activation (Figure 1, Table 1), which results in increased generation and release of BDNF (i.e., a synaptic growth factor associated with memory and learning) in the central nervous system [51]. Bryostatin 1 also causes activation of the non-amyloidogenic, α-secretase processing pathway of amyloid precursor protein [52]. Preclinical studies revealed that intraperitoneal bryostatin 1 administration activates PKCε in the brain and prevents Aβ accumulation, synaptic loss, and memory deficit in AD transgenic mouse models [53,54]. Furthermore, bryostatin 1 mediates preservation of synapses and ameliorates memory in both aged rat models and rodent models of Fragile X syndrome and stroke [55,56,57]. It has also been reported that oral administration of bryostatin ameliorates learning and memory in an AD mouse model [58]. Bryostatin also ameliorates neurological decline and anti-inflammatory immune response in a multiple sclerosis mouse model [59].

#### 3.1.2. Clinical Evidence

It was reported through a phase IIa clinical study that a single intravenous administration of bryostatin elevated the Mini-Mental State Examination (MMSE) score of six AD individuals in comparison with three placebo-receiving individuals [34]. Indeed, bryostatin was found to be well-tolerated in these patients with AD and no significant bryostatin-associated adverse effects were observed. Elevated peripheral blood mononuclear cells PKCɛ levels were detected after one hour of infusion, along with peak concentrations of bryostatin in blood. Prolonged treatment with bryostatin triggered PKCɛ downregulation that was reliant on the duration of treatment and dosing levels. A significant downregulation of PKCɛ was observed when maximum doses (25 μg/m^2^) were administered for five of six consecutive weeks [34]. This study also indicated that bryostatin might be an effective drug candidate in AD treatment. In a different phase II clinical study, bryostatin showed improved efficacy, tolerability, and safety when administered in 150 patients with advanced AD to ameliorate loss of cognitive functions [60]. Interestingly, no significant primary endpoint was attained in the full analysis set (FAS). However, pre-specified and post hoc exploratory analyses as well as primary and secondary analyses in the completer analysis set (CAS) revealed positive outcomes in the bryostatin (20 μg)-treated group in comparison with the placebo group. Collectively, these findings suggest further clinical studies are needed to determine the efficacy of bryostatin (20 μg) in AD treatment [60]. A different phase II trial was started in June 2018 among 108 patients with AD (who were not receiving memantine). In that study, patients were divided into two groups according to their MMSE scores, 10–15 versus 4–9. Subsequently, these groups were randomized to receive either placebo or bryostatin (20 μg). In total, seven doses were administered over twelve weeks [61]. Unfortunately, the results of this study were not satisfactory.

### 3.2. Homotaurine

#### 3.2.1. Preclinical Evidence

Homotaurine (tramiprosate) is a natural amino acid found in several species of red marine algae [62]. This compound is analogous to taurine, though containing an additional carbon in its chain. It has been revealed by preclinical studies that tramiprosate decreases the formation of Aβ oligomers and the deposition of amyloid fibrils as plaques in an AD mouse model. Tramiprosate treatment reduced the concentrations of soluble amyloid proteins and the deposition of amyloid plaques in the brain [63]. In a dose-dependent manner, plasma levels of Aβ were declined, which further suggests the contribution of tramiprosate to brain Aβ metabolism or Aβ transport [63]. A preclinical study reported that tramiprosate mediates polymerization of tau in fibrillar aggregates; however, these aggregates of tau did not exert any toxic effects in neuronal cell cultures. Moreover, tramiprosate did not influence the binding of tau with microtubules, rather it mediated the reduction of tau-actin complexes that might be toxic for the cells [64]. 

#### 3.2.2. Clinical Evidence

In a phase II clinical trial, it was confirmed that tramiprosate safely decreases concentrations of Aβ42 in the cerebrospinal fluid (CSF) of individuals with mild-to-moderate AD. Indeed, these decreased levels of Aβ42 reported in CSF and long-term clinical observations, which suggests a role for tramiprosate in disease modification. Furthermore, after a three month treatment, tramiprosate was found to be well-tolerated and safe [65]. Although tramiprosate did not exhibit marked differences in the subsequent phase III study (Alphase study), the findings of this study were inexplicably variable [66]. Furthermore, a pooled analysis of the two phase III trials among 2025 mild-to-moderate AD patients (considering the distribution of ApoE4 allele) revealed a positive trend in Clinical Dementia Rating Scale Sum of Boxes scores (CDR-SB) and marked differences in ADAS-cog scores in homozygote individuals (who received 150 mg two times a day). Interestingly, non-*APOE4* individuals did not show any clinical benefits, and *APOE4* heterozygotes exhibited an intermediate level of efficacy [67]. Subsequent re-analyses of these data showed most efficacies in the homozygote individuals, who were at the mildest clinical stage of disease (MMSE, 22–26). In the case of those individuals, tramiprosate exhibited benefits on disability assessment for dementia (DAD), CDR-SB, and ADAS-cog in comparison with the placebo group. Cognitive stabilization was detected in ADAS-cog over 78 weeks, whereas functional (DAD) and cognitive (ADAS-cog) effects were elevated over time [68].

In a subgroup of patients (*n* = 312), tramiprosate’s effect on the volume of hippocampus was assessed in the Alphase study. It was confirmed that there is an important relationship between the dose of tramiprosate and the reduced change levels in hippocampal volume [69,70]. Collectively, these findings indicate the disease-modifying role of tramiprosate in cases of AD at the earliest AD clinical stages, particularly for the patients containing ApoE4/4 [68].

### 3.3. Anabaseine and Its Derivative GTS-21

#### 3.3.1. Preclinical Evidence

Anabaseine (3,4,5,6-Tetrahydro-2,3’-bipyridine) is an alkaloid toxin generated via nemertines, which is a phylum of carnivores (particularly marine worms) [71]. GTS-21 (a synthetic anabaseine derivative) has been found to play a role as a partial agonist in neural nicotinic acetylcholine receptors [72]. Indeed, anabaseine can act as a potent agonist at the level of neurons and muscle alpha-bungarotoxin-sensitive nicotinic receptors (Figure 1, Table 1) [73]. 

Nicotinic acetylcholine receptors (nAChRs) are ionotropic cholinergic receptors that are sensitive to activation by nicotine. GTS-21 has the capacity to bind with both the α7 and α4β2 subtypes, while it significantly activates α7 subtypes [74,75]. The main brain regions for α7 nAChR expression include the hippocampus and the pre- and frontal cortex. Furthermore, α7-nAChR is associated with important cognitive functions, including judgment, language, orientation, calculation, learning capacity, comprehension, thinking, and memory. It was found that Aβ binds with α7-nAChR to induce receptor inhibition or activation in an Aβ concentration-dependent manner. Aβ oligomers also induce phosphorylation of tau via activation of α7-nAChR. Therefore, agonists of α7-nAChR and/or positive allosteric modulators of α7-nAChR might be effective in AD treatment [76]. 

#### 3.3.2. Clinical Evidence

Kitagawa et al. [77] have determined the effects, pharmacokinetics, tolerability, and safety of GTS-21 on cognitive functions in healthy male subjects. In total, 18 subjects were randomized to receive either placebo or GTS-21 at doses of either 150, 75, or 25 mg (three times a day during the first four days, once on the fifth day) for three, five-day sessions. At doses up to 450 mg/day, GTS-21 was found to be well-tolerated, along with no clinically important safety issues. The area under the plasma drug concentration and C_max_ of GTS-21 and its metabolite, 4-OH-GTS-21, were increased in a dose-dependent manner; however, a substantial intersubjective variability was observed, though being reduced with continuous dosing. GTS-21 exerted a statistically significant improvement in cognitive function, including episodic secondary memory, working memory, and attention, as compared with placebo. In addition, a link between GTS-21 exposure and the extent of the cognitive response was observed, with a maximum effect being approached for doses between 150 and 75 mg three times a day. Collectively, these findings suggest that GTS-21 might be effective as a new treatment for dementia [77].

### 3.4. Rifampicin 

#### 3.4.1. Preclinical Evidence

Rifamycin is a broad-spectrum antibiotic that was formerly derived from *Amycolatopsis* (a species of Gram-positive bacteria). Furthermore, rifamycin is also obtained from *Salinispora* (a marine bacterium) extracted from *Pseudoceratina clavata* (a marine sponge) [78]. In addition to its conventional anti-infectious activity, rifampicin has displayed substantial neuroprotective activity in several experiments. It also reduces free radical injury and neuroinflammation, which further results in marked neuroprotective activity (Table 1) [79]. The generation of free radicals also plays a significant role in Aβ production [80,81]. Various studies have already shown the therapeutic activity of antioxidants in Aβ plaque-associated neurotoxicity in AD. The study of Tomiyama et al. [82] confirmed that rifampicin suppresses fibril formation and aggregation of synthetic Aβ_1–40_ and averts neurotoxic effects in pheochromocytoma PC12 rat cells in a dose-dependent manner. Rifampicin was found to be 10–100 times more effective as compared with vitamins in suppressing Aβ aggregation. A different study revealed that rifampicin’s ansa-chain is not required for suppressing Aβ aggregation, whereas its lipophilicity plays a significant role in the transport of drugs into the brain in vivo [83]. Various studies carried out in vitro have illustrated rifampicin’s anti-amyloid activity, including suppression of the amyloid fibril formation [84,85]. A number of studies also evaluated the rifampicin’s anti-amyloid activity on the aggregation of amylin fibrin and associated toxicity, showing that the observed suppressive activity was mediated through rifampicin binding with peptide fibrils instead of their possible intracellular antioxidant effect [86,87,88]. 

The study of Umeda et al. [89] has shown that rifampicin exerts marked effects against the buildup of tau oligomers and Aβ in multiple transgenic mouse models. Rifampicin treatment for one month significantly decreased tau and amyloid toxicity linked with ameliorated microglial activation and synapse loss. In addition, rifampicin ameliorated memory loss and suppressed apoptotic pathways, such as by activating caspase 3 and releasing cytochrome c in the hippocampus [89]. Rifampicin also induced the restoration of autophagy-lysosomal activity. Despite some slight differences in Aβ deposition in various transgenic mouse models (including AD, amyloid oligomers, and tauopathy model), findings suggested that rifampicin shows marked inhibitory effects on apoptotic pathways, microglial activation, hyperphosphorylation of tau, and accumulation of tau and Aβ oligomers—all these effects being positively linked with neurocognitive outcomes [89]. Collectively, these findings indicate rifampicin’s therapeutic potential as a neuroprotective agent in cases of AD. It has also been clearly reported that impaired Aβ clearance across the blood–brain barrier (BBB) may result in the formation of Aβ deposits in the brain and the related progression of AD [90,91,92]. Interestingly, caffeine and rifampicin increased Aβ clearance from the brain via upregulating low-density lipoprotein receptor-related protein 1 (LRP1) and P-glycoprotein (P-gp) at the BBB [93]. These findings suggest the existence of a probable receptor/transporter that has a significant contribution in the clearance of Aβ, which was found to be elevated by rifampicin [94]. In a rat dementia model, Kaur et al. [95] showed that rifampicin markedly ameliorated locomotor damage and memory deficit. 

#### 3.4.2. Clinical Evidence

In contrast with preclinical results, only a small number of clinical studies have assessed the activity, efficacy, and outcomes of rifampicin in AD patients. In 140 Japanese non-demented elderly volunteers, Namba et al. [96] evaluated 16 brains from leprosy patients without dementia and compared the senile plaques and neurofibrillary tangles by immunohistochemical staining. As compared with age-matched controls, their study revealed that elderly non-demented leprosy patients who received rifampicin exhibited an abnormal absence of senile plaques in their brain [96]. Unfortunately, these findings have not been replicated in subsequent studies, which further suggests that rifampicin does not influence AD prevalence in patients with leprosy [97]. Follow-up studies were designed to reveal rifampicin’s causal anti-dementia action, but they failed to provide clear clinical findings. In 101 patients with mild-to-moderate AD, Loeb et al. [98] demonstrated the anti-dementia effects of rifampicin (oral administration of 300 mg/day for three months), resulting in a marked enhancement of cognitive function via a measurement using a standardized ADAS-Cog (SADAS-cog) score. However, these auspicious findings could not be confirmed in a study involving a rifampicin treatment for 12 months [99]. 

In another study, Iizuka et al. [100] showed that the preventive activity of rifampicin requires a minimum dose of 450 mg/day for one year, even for the period of predementia. These authors also revealed in a retrospective fluorodeoxyglucose (FDG)-positron emission tomography (PET) study that a treatment with rifampicin markedly ameliorated cognitive and metabolic (posterior cingulate gyrus) deficits at the predementia stage in the long-term follow up. At the dose of 450 mg/day for over one year, rifampicin treatment considerably ameliorated the uptake of FDG in the posterior cingulate gyrus region, which was also reflected in the MMSE scores.

Oral administration of rifampicin resulted in infrequent adverse events, including liver damage in humans, which renders its prolonged use more difficult. Considering the noninvasiveness and ease of rifampicin administration, intranasal administration might be the best approach for a prolonged administration of rifampicin [101].

### 3.5. Dictyostatin 

#### 3.5.1. Preclinical Evidence

Dictyostatin (a marine-derived macrolide) was first extracted from *Spongia* sp. (a Maldives marine sponge) [102]. It has been reported that tau is aberrantly hyperphosphorylated in case of AD [103]. Makani et al. [104] estimated the efficacy of dictyostatin in a PS19 tau Tg mouse model. It was observed that dictyostatin-treated PS19 mouse models showed ameliorated density of microtubules and decreased levels of axonal dystrophy (Figure 1) along with a decreased level of tau pathology and a tendency toward an elevated survival rate of hippocampal neurons, as compared with vehicle-treated PS19 mouse models [104]. In dictyostatin-treated aged PS19 mouse models, the practical positive results obtained on the brain effect reinforced the idea that microtubule-stabilizing molecules might be effective in AD treatment.

#### 3.5.2. Clinical Evidence 

In a clinical trial, the effects of rifampicin and doxycycline were evaluated in 101 individuals with mild-moderate dementia and probable AD [98]. In that study, it was suggested that treatment with rifampicin and doxycycline might have beneficial effects in mild-moderate AD patients [98]. In order to refute or confirm these results, a different clinical study was also carried out [105]. A marked deterioration was observed in SADAS-cog over time with doxycycline and rifampicin as compared with placebo. Collectively, no statistically significant deterioration/decline was observed as compared with placebo (*n* = 305). Furthermore, there was no marked effect of either doxycycline or rifampicin on Clinical Dementia Rating Scale Sum of Boxes scores (CDR-SB). Similar patterns were also observed in secondary outcomes. This study concluded with the fact that treatment with rifampicin or doxycycline for one year (alone or in combination) had no beneficial outcomes on function or cognition in AD [105].

### 3.6. Anhydroexfoliamycin and Undecylprodigioisin 

Anhydroexfoliamycin and undecylprodigioisin are derived from *Streptomyces,* and these compounds were formerly found to exert antioxidant activities. In an oxidative damage model, their effects were demonstrated in primary cortical neurons, suggesting their capacity to decrease the levels of reactive oxygen species (ROS) and to improve the antioxidant defenses via elevation of the glutathione levels and catalase activity. Nevertheless, only anhydroexfoliamycin was demonstrated to be an inductor of nuclear factor erythroid 2–related factor 2 (Nrf2) [106]. Moreover, these two compounds play a role in mitochondria by decreasing the caspase-3 effect and preserving the mitochondrial membrane potential (MMP), whereas undecylprodigioisin solely improves mitochondrial activity, and anhydroexfoliamycin regulates calcium homeostasis [106]. As oxidative damage is strongly associated with neurodegenerative disorders, Leirós et al. [107] evaluated the effects of both compounds on the principal hallmarks of AD. Moreover, their effects were assessed in vitro in a tau model for AD (SH-SY5Y-TMHT441) and APP metabolic studies in BE(2)-M17 cells. In addition, expression of the glycogen synthase kinase-3beta (GSK3β), the extracellular signal-regulated kinase (ERK), tau phosphorylation, β-secretase effects, and Aβ levels were studied. Although undecylprodigiosin showed poor outcomes, anhydroexfoliamycin significantly suppressed GSK3β (Figure 1) and decreased tau phosphorylation in vitro at a dose of 0.1 μM. It was found by using SP600125 (a specific inhibitor of c-Jun N-terminal kinase (JNK)) and a competitive assay of anhydroexfoliamycin that the decreased levels of phosphorylated tau in SH-SY5Y-TMHT441 cells were facilitated by the JNK signaling cascade. Anhydroexfoliamycin activity was evaluated in vivo via intraperitoneal administration in 3xTg-AD mouse models, which confirmed the positive outcomes observed with in vitro studies [107]. Collectively, these findings suggest that anhydroexfoliamycin might be an effective drug candidate for treating AD, though this should be further confirmed.

### 3.7. Gracilins

**Figure 2:**
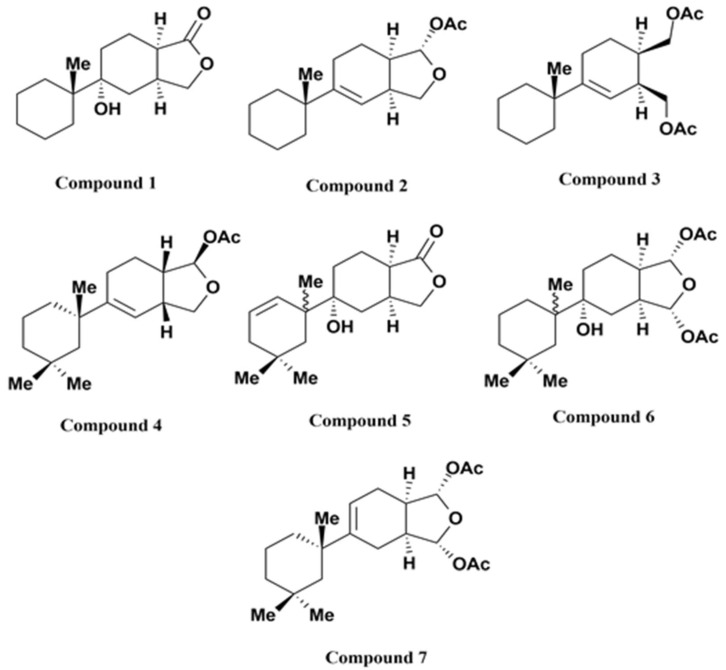
Chemical structures of the gracilin A derivatives.

Gracilins are diterpenoid compounds derived from *Spongionella gracilis*, a marine sponge [108]. It has been reported that these diterpenoid compounds exert anti-inflammatory properties as inhibitors of phospholipase A2 (PLA2, Table 1) [109]. Gracilins exert neuroprotective and antioxidant properties via inducing Nrf2 and targeting mitochondria [110]. Oxidative damage is associated with mitochondrial impairment and thus with neurodegenerative diseases. This possible neuroprotective activity of gracilins suggests that they may be important lead candidates in anti-AD drug development. Derivatives of gracilin A have been synthesized through a pharmacophore-directed retrosynthesis (PDR) approach and found to contain strong neuroprotective properties. Abbasov et al. [111] confirmed that gracilin A derivatives (compounds 2, 3, 4, and 7; Figure 2) provided protection to SH-SY5Y cells against hydrogen peroxide-mediated injury via recovering GSH content, reducing reactive oxygen species (ROS) levels, improving MMP, and elevating cell survival. In a different study, the activity of the gracilin A derivatives (compounds 1–7) was evaluated [112]. The capacity of these compounds to regulate the expression of various antioxidant genes, including Nrf2, superoxide dismutase, glutathione peroxidase, and catalase, were estimated in SH-SY5Y cells. Among all the gracilin A derivatives tested, compounds 2 and 3 were the most effective as lead compounds for AD. Additionally, the anti-neuroinflammatory potentials of all these derivatives were evaluated in lipopolysaccharide (LPS)-activated BV2 microglial cells. Numerous derivatives reduced the secretion of various cytokines, including tumor necrosis factor-α, granulocyte-macrophage colony-stimulating factor, interleukin-6, and interleukin-1β and other harmful molecules (such as nitric oxide, ROS). These derivatives also controlled the translocation of NF-κB and Nrf2 and decreased the activation of p38. These protective activities were demonstrated in a trans-well coculture with SH-SY5Y and BV2 cells, and multiple derivatives elevated SH-SY5Y survival [112]. 

### 3.8. 13-Desmethyl Spirolide-C

13-Desmethyl spirolide C (SPX; a marine compound, Figure 1) accumulates in shellfish; this compound belonging to the cyclic imine group is derived from the marine dinoflagellate *Alexandrium ostenfeldii* [113]. Using a triple transgenic mouse model (3xTg) for AD, Alonso et al. [114] evaluated the action of SPX in tau hyperphosphorylation and Aβ accumulation. SPX treatment decreased intracellular accumulation of Aβ and phosphorylated tau levels in 3xTg cortical neurons in vitro (Table 1). Treatment with SPX did not influence the steady-state levels of M1 and M2 muscarinic and α7 nicotinic acetylcholine (ACh) receptors, whereas it reduced the extent of acetylcholine-mediated effects and elevated levels of ACh in 3xTg neurons. In addition, the use of SPX reduced the concentrations of two protein kinases associated with ERK, GSK-3β, and tau phosphorylation. SPX also eliminated the glutamate-mediated neurotoxic effects in control and 3xTg neurons [114]. An in vivo study also reported decreased levels of intracellular Aβ [115]. It was shown that the intraperitoneal administration of SPX (11.9 µg/kg) mediated positive outcomes on AD markers along with raised levels of N-acetyl aspartate (NAA). These findings were further supported by an observed rise in the levels of synaptophysin and a reduction of intracellular Aβ levels in the hippocampus of treated-3xTg-AD mouse models versus non-treated mouse models, which suggests positive outcomes of SPX in a well-known AD model. These findings suggest that SPX can cross the blood–brain barrier and can exert beneficial effects in vivo against AD after intraperitoneal administration of low doses of SPX [115]. Therefore, SPX might be effective and possibly used for novel AD treatment. 

### 3.9. Docosahexaenoic Acid 

#### 3.9.1. Preclinical Evidence

In the brain, docosahexaenoic acid (DHA) is the most plentiful long-chain polyunsaturated fatty acid [116]. Low DHA levels mediate various AD characteristics, whereas normal or increased concentrations avert or improve them. Even though DHA can be generated from plant-derived ω-3 fatty acids, this metabolic pathway is ineffective in humans. In the human brain, most DHA comes from marine foods and supplements [117]. DHA supplementation decreased Aβ plaque generation, toxicity, and aggregation and mediated clearance of Aβ plaque in individuals with AD and moderate dementia [118,119,120]. DHA supplementation also reduced the levels of tau tangles. Animal studies confirmed that supplementation with DHA reduced tau pathology [121]. Since DHA is a vital constituent of cell membranes, it therefore mediates glucose delivery into the brain via controlling GLUT1 transporters. As compared with nonhuman primate and rodent controls, deficiency of DHA reduced GLUT1 transporters in rat models by up to 30% [122,123]; however, supplementation with DHA elevated GLUT1 transporters by 37% and caused endothelial cells to take up more glucose [124,125]. 

#### 3.9.2. Clinical Evidence 

DHA supplementation (∼2 g) for six months showed a reduced level of phosphorylated tau protein in the CSF of AD patients [126]. In a clinical study, a DHA supplement (containing fish oil formulation named EPAX 1050 TG) was administered for six months to 204 individuals with mild-to-moderate AD; among them, 174 individuals completed the study. The placebo and treatment-receiving groups did not vary on either of the key outcome measures, including decline on the ADAS-cog and MMSE scores or on neuropsychiatric symptoms in general. Nonetheless, it was suggested by a subgroup analysis of the 32 mildest cases that there was less of a decline in MMSE score (however, not ADAS-cog score), and a comparable reduced decline seemed to take place in the placebo group once swapped with DHA supplement after 6 months [127,128]. Moreover, it was indicated by substudies that DHA supplementation elevated CSF levels of DHA and other fatty acids and reduced tau levels and altered expression of inflammation-associated genes and secretion of specific cytokines in white blood cells [126,129,130]. In a North American AD Cooperative Study (conducted at 51 centers), DHA treatment was provided to 402 individuals with mild-to-moderate AD for 18 months, among them 295 individuals finished the trial. As compared with placebo, DHA had no activity on the extent of decline on either the ADAS-cog or Clinical Dementia Rating Scale Sum of Boxes (CDR-SB) scores. A study on the participants with the ApoE genotype suggested a decreased cognitive deficit in ApoE4 noncarriers, who might have had comparatively less advanced AD [116]. 

The DHA clinical trial called the Multidomain Alzheimer Preventive Trial (MAPT) was carried out in four cities in France. In this trial, a secondary prevention study was carried out in 1,680 volunteers; among them were people 70 and older who stated a mild functional loss and subjective memory complaint. They were also weak and walked slowly; however, they did not meet an AD diagnosis [131]. In MAPT, three interventions including DHA (800 mg) and eicosapentenoic acid (EPA) (225 mg) alone on a daily basis, multidomain behavioral intervention alone, and DHA/EPA as well as a multidomain behavioral intervention were compared with placebo [132]. None of the interventions (alone or in combination) markedly reduced cognitive deficit as assessed via a composite score of four tests: verbal fluency, processing, orientation, and recall [133]. In an amyloid PET substudy, the multidomain intervention (without or with DHA/EPA) ameliorated composite scores after 3 years as compared with placebo in the amyloid positive (but not negative) subset, but DHA/EPA alone exerted no action in either subset [134]. A decreased level of cortical amyloid was observed after 2 years in individuals who received the multidomain intervention (without or with DHA/EPA); however, a similar effect was not observed in individuals who received DPA/EPA only [135].

**Table 1:** Various bioactive compounds derived from marine sources showed beneficial effects in AD treatment.

Compound	Marine Source	Mechanism of Action	Results of Animal Studies	Outcomes of Clinical Studies	References
Bryostatin-1	*Bugula neritina*	Enhances spatial learning and memory; improves cognitive function and activities of daily living in moderate-to-severe AD; decreases Aβ level; retrieves neurotrophic activity; modulates neuronal synapses under synaptic dysfunctions	Activated PKCε in the brain and prevented Aβ accumulation of synaptic loss and memory deficit in AD transgenic mouse models; mediated preservation of synapses and improved memory in aged rat models; improved learning and memory in an AD mouse model	Increased the Mini-Mental State Examination (MMSE) score of AD patients	[34,43,53,54,58,136,137]
Homotaurine (tramiprosate)	Seaweed	Activates sirtuin 1; decreases the formation of Aβ oligomers and deposition of amyloid fibrils as plaques	Reduced the formation of Aβ oligomers and deposition of amyloid fibrils as plaques in an AD mouse model	Safely decreased the levels of Aβ42 in the cerebrospinal fluid (CSF) of individuals with mild-to-moderate AD	[63,65]
Anabaseine	Ribbon worm (*Amphiporus* sp.)	Potent agonist of alpha-bungarotoxin-sensitive nicotinic receptors; improves memory	Stimulated nicotinic acetylcholine receptors (especially at the neuromuscular junction) in various animal models	GTS-21 (a synthetic anabaseine derivative) exerted a statistically significant improvement in cognitive function, including episodic secondary memory, working memory, and attention	[71,73,77,138]
Rifamycin	*Pseudoceratina clavata*	Reduces neuroinflammation and free radical injury; exerts significant neuroprotective activity; suppresses fibril formation and aggregation of Aβ_1–40_; suppresses Aβ aggregation	Inhibited fibril formation and aggregation of synthetic Aβ_1–40_ and prevented neurotoxic effects in pheochromocytoma PC12 rat cells in a dose-dependent manner; exerted marked effects against the buildup of tau oligomers and Aβ in multiple transgenic mouse models	Exhibited an abnormal absence of senile plaques in the brains of leprosy patients; anti-dementia effects in 101 patients with mild-to-moderate AD	[79,83,89,96,98]
Dictyostatin	*Spongia* sp.	Improves density of microtubules; reduces levels of axonal dystrophy and tau pathology; increases survival rate of hippocampal neurons	Improved density of microtubules and reduced levels of axonal dystrophy along with a decreased level of tau pathology and a tendency towards an elevated survival rate of hippocampal neurons in mouse models	Exerted beneficial effects in mild-moderate AD individuals; showed no significant effect in case of Clinical Dementia Rating Scale Sum of Boxes (CDR-SB)	[98,104,105]
Anhydroexfoliamycin and undecylprodigioisin	*Streptomyces*	Exert antioxidant properties; decrease the levels of reactive oxygen species (ROS); elevates the glutathione levels and catalase activity; induces nuclear factor erythroid 2–related factor 2 (Nrf2); reduces the caspase-3 effect; preserves the mitochondrial membrane potential (MMP)	Undecylprodigiosin exerted poor outcomes; anhydroexfoliamycin significantly suppressed GSK3β and decreased tau phosphorylation	-	[106,107]
Gracilins	*Spongionella gracilis*	Shows anti-inflammatory activities; neuroprotective and antioxidant properties; suppresses BACE-1; decreases tau phosphorylation;	Provided protection to SH-SY5Y cells against hydrogen peroxide-induced injury via reducing reactive oxygen species (ROS) levels, recovering GSH content, improving MMP, and elevating cell survival; regulated the translocation of NF-κB and Nrf2 and reduced the activation of p38 in SH-SY5Y and BV2 cells	-	[109,110,111,139]
13-Desmethyl spirolide C	*Alexandrium ostenfeldii*	Decreases intracellular Aβ accumulation and levels of hyperphosphorylated tau; reduces intracellular levels of Aβ	Reduced intracellular accumulation of Aβ and phosphorylated tau levels; decreased acetylcholine-mediated effects and elevated ACh levels; reduced the levels of 2 protein kinases linked with ERK, GSK-3β, and tau phosphorylation; eliminated the glutamate-induced neurotoxic effects; mediated positive outcomes on AD markers; increased levels of N-acetyl aspartate	-	[113,114,115]

## 4. Conclusions

There is a substantial need for safe, effective, and novel treatments for AD. Natural products derived from marine organisms have the potential to serve as an excellent source that can be used to expand the pharmaceutical pipeline. Various novel compounds derived from marine organisms have exhibited significant effects in several in vivo and in vitro studies against AD pathogenesis. Research shows that nature is a great source of compounds that can be used for AD treatment. It is now feasible to develop effective bioactive compounds from marine sources because of the technological advances made in harvesting samples and because of advances in the purification and characterization of the products. Therefore, more studies are required on marine organisms to develop novel and effective therapeutic agents to treat AD. 

## Data Availability

Not applicable.
